# Design and Preliminary Validation of Individual Customized Insole for Adults with Flexible Flatfeet Based on the Plantar Pressure Redistribution

**DOI:** 10.3390/s21051780

**Published:** 2021-03-04

**Authors:** Yangzheng Jiang, Duojin Wang, Jiming Ying, Pengfei Chu, Yu Qian, Wenming Chen

**Affiliations:** 1 School of Medical Instrument and Food Engineering, University of Shanghai for Science and Technology, Jungong Rd. 516, Shanghai 200093, China; 192672193@st.usst.edu.cn (Y.J.); 182702207@st.usst.edu.cn (J.Y.); 193832315@st.usst.edu.cn (Y.Q.); 2 Shanghai Engineering Research Center of Assistive Devices, Jungong Rd. 516, Shanghai 200093, China; 3 Academy for Engineering & Technology, Fudan University, Handan Rd. 220, Shanghai 200433, China; pfchu18@fudan.edu.cn (P.C.); chenwm@fudan.edu.cn (W.C.)

**Keywords:** flatfoot, insole, plantar pressure redistribution, 3D modeling, gait

## Abstract

Flatfoot is a common musculoskeletal deformity. One of the most effective treatments is to wear individually customized plantar pressure-based insoles to help users change the abnormally distributed pressure on the pelma. However, most previous studies were divided only into several plantar areas without detailed plantar characteristic analysis. In this study, a new insole is designed which redistributes pressure following the analysis of characteristic points of plantar pressure, and practical evaluation during walking of subjects while wearing the insole. In total, 10 subjects with flexible flatfeet have participated in the performance of gait experiments by wearing flat insoles, orthotic insoles, and plantar pressure redistribution insoles (PPRI). The results showed that the stance time of PPRI was significantly lower than that of the flat insoles under slow gait. PPRI in the second to third metatarsal and medial heel area showed better unloading capabilities than orthotic insoles. In the metatarsal and heel area, the PPRI also had its advantage in percentage of contact area compared to flat insole and orthotic insole. The results prove that PPRI improves the plantar pressure distribution and gait efficiency of adults with flexible flatfeet, and can be applied into clinical application.

## 1. Introduction

The foot is an indispensable organ for human walking and one of the most important sensory organs of the human body. Improper use of the foot will lead to an irreversible change in its shape, which affects the plantar pressure to varying degrees [1]. Flatfoot (pes planus) is a common musculoskeletal deformity, which is mainly manifested by the collapse of medial longitudinal arch of foot [2], and its occurrence is related to age, gender, and weight [3]. The arch is an important structure of the human foot. Its elasticity plays an essential role in cushioning oscillations during walking and jumping, and it guarantees the support and stability of the body when standing upright [4]. 

Normal foot pronates in the initial stance phase of the normal gait, then supinates to accommodate and absorb the impact of the foot when it in touch with the ground [5]. For sarapus, due to the absence of the arch, the foot will have a rapid pronation after the initial stance phase, and there will be an extended pronation by the foot in touch with the ground [6,7]. The abnormal performance of the flatfoot during the gait cycle will increase the pressure inside the foot and also raise the risk of body damage and injury [8].

There are two types of flatfoot: flexible and rigid [9]. The arch of the flexible flatfoot will disappear under weight-bearing conditions, but it will recover in a non-weight-bearing state. In this case, the joint mobility of the foot still exists; while the arch of the rigid flatfoot will collapse or disappear, regardless of whether it is weight-bearing [10]. Flexible flatfeet tend to evolve into rigid flatfeet over time [9]. Conservative physical therapy can reduce the probability of this development, which is essential for early detection and appropriate management in flexible flatfeet [11]. Foot orthoses can improve foot deformities by limiting the collapse of the arch. Studies have shown that adults with sole pain due to flatfeet can improve their standing and walking posture by wearing foot orthoses, and thereby relieve pain [12].

In addition to wearing foot orthoses, traditional orthopedic insoles can also relieve the symptoms of the most flatfoot patients and delay the progression of the disease, but there are still limitations: although sarapus generally show a certain consistency due to the arch collapse, there are still huge differences in the distribution of plantar pressure between sarapus [1]. If the orthopedic insoles cannot adapt well to the individual’s foot characteristics, their irregular use may even damage the lower limbs or aggravate foot deformities [13]. Therefore, a customized orthopedic insole following the characteristics of the sarapus can reduce arch collapse and improve plantar pressure distribution.

Some previous studies have confirmed that customized insoles can be used as an auxiliary physiotherapy tool, which has a positive effect on helping subjects change their gait and re-adjust the pressure distribution on the pelma [14,15,16,17]. Designing customized insoles based on plantar pressure can specifically help users change the abnormally distributed pressure on the plantar, which is one of the most common traditional methods. The lower limb movement system of the human body is a chain structure, and the posture changes of the ankle during walking will also change the movement mode of the knee joint and hip joint of the lower limb. From a biomechanical point of view, customized insoles according to the patient’s plantar pressure distribution can promote the correction of foot deformities by changing the ankle movement posture, and effectively reduce the load on the lower limbs [18].

Due to the abnormal foot shape of individuals with flexible flatfeet, their walking pattern will be different from that of normal people, which causes abnormally higher pressure in some areas of the sole of the foot [19,20]. Foot deformity can cause abnormal plantar pressure distribution in individuals with flexible flatfeet, and effective analysis of the plantar pressure in the flatfoot individual’s gait cycle can also directly or indirectly understand the condition of the foot deformity [21]. Therefore, the analysis of plantar pressure helps for a better design of customized insoles [16]. This is also a necessary step for insole customization for individuals with flatfeet.

In addition, the pressure difference in different areas of the soles of the subjects will affect their comfort during walking. The higher pressure on the heel and the second to third metatarsals in gait leads to discomfort these areas rather than other areas of the sole [22]. Each part of the human foot has a specific biomechanical function [23]. The reasonable operation of the foot function can be ensured only when the pressure in each area is within the normal range. Appropriate distribution of the pressure in each area of the sole can effectively avoid the damage to certain high-pressure areas of the sole [24]. Therefore, scientific analysis of plantar pressure distribution can help prevent plantar injury to a greater extent [25]. At present, many researches take plantar pressure into account when they design customized insoles, but they basically divide insoles into several regions and pay more attention to arch area. The above method can be improved in the following two aspects: firstly, the precision of plantar pressure data can be improved by increasing the density of plantar pressure sensor; furthermore, the rest area of the sole needs to be concerned beside the arch area.

Thus, in the present study, we studied the plantar pressure following the two aspects and designed plantar pressure redistribution insoles (PPRI) for adults with flexible flatfeet, and compared them with ordinary orthotic insole and flat insole. The gait parameters of level walking at different gait speed, including stance time, step rate, peak pressure, contact area of the foot are evaluated. 

## 2. Materials and Methods

### 2.1. Equipment

**Figure 1:**
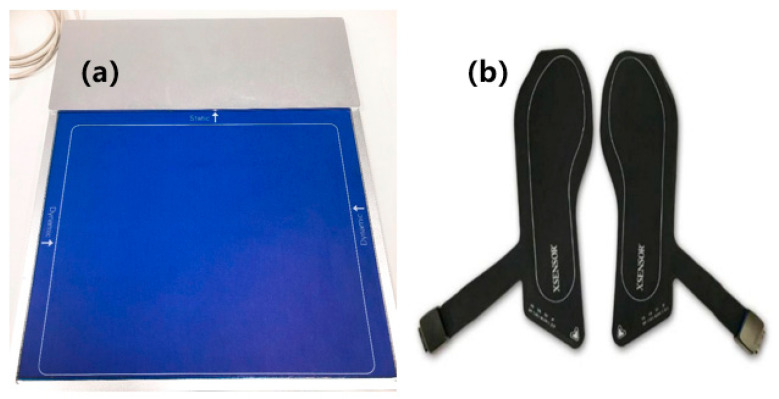
(**a**) Self-designed plantar pressure test plate; (**b**) Wireless insole.

A self-designed plantar pressure test plate was used to diagnose the flat arch index (AI) for obtaining the plantar pressure data of the subject which are used for the design of the height of each characteristic point on the surface of the insole. The foot pressure plate is composed of an array type thin film pressure sensor (42 × 52 sensor array), as shown in Figure 1a. Each piezoresistive membrane pressure sensor was in accordance with the pressure resistance linear correlation within the range of use, and the weight of the subject was equal to the total pressure of all the pressure sensors, so the stress could be calculated by the ratio of the resistance value of each membrane pressure sensor to the total resistance. To sum up, we only needed to ensure the accurate weight of the subjects without calibration, and could also ensure the accuracy of plantar pressure data.

A wireless insole type and plantar pressure analysis system (Xsensor X4, Calgary, AB, Canada) was used to provide complete data recording and analysis functions, including real-time browsing, storage, and analysis of data, in addition to plantar pressure analysis functions, as well as gait analysis function. The wireless pressure insole is placed on the experimental insole, orthopedic insole, and flat insole. The sampling rate of each subject is 150 fps while walking. A single wireless pressure insole consists of 230 individual capacitive pressure sensors, the pressure range of each single sensor is 1–128 psi, as shown in Figure 1b.

### 2.2. Design of PPRI

**Figure 2:**
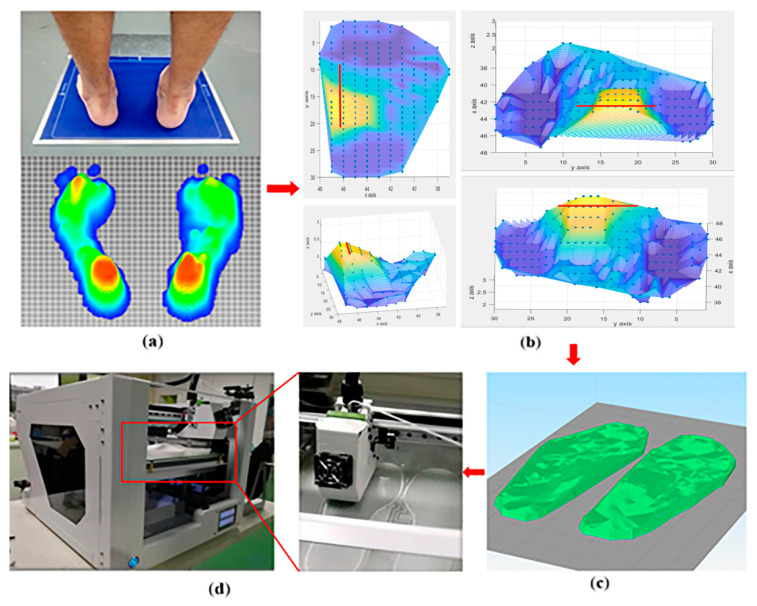
Design process of PPRI. (**a**) Characteristic point data collection of plantar pressure; (**b**) 3D map of insole surface characteristic point cloud; (**c**) Insole software model preview; (**d**) PPRI 3D printing.

Firstly, subjects stand still on the plantar pressure test plate. The plantar pressure data from subjects were collected from four separated areas of the sole, including toe (T), metatarsals (M), midfoot (MF), and heel (H), as shown in Figure 2a. The x, y value of each three-dimensional coordinate point represents the location of the foot on the plantar pressure test plate, and the *z*-axis represents the value of the pressure. The value of pressure is inputted to calculate the height of the characteristic points on the surface of the insole, as shown in Figure 2b. Then the insole model was generated through software modeling, as shown in Figure 2c. Finally, the plantar pressure redistribution experimental insole was obtained by 3D printing, as shown in Figure 2d. 

**Figure 3:**
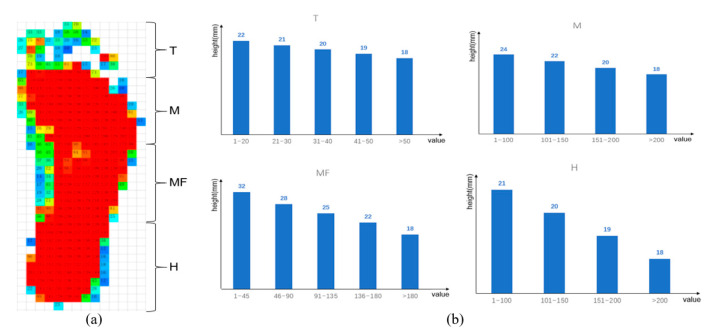
Conversion of pressure distribution to height information in each area (including T, M, MF, and H area). (**a**) Original pressure value. (**b**) Redistribution plan for the pressure value of four plantar areas.

The pressure redistribution from the plantar pressure into the height of the characteristic points on the surface of the insole is calculated as follows. Take MF area as an example. Since the arch height of a normal person is 12–16 mm, the height difference of MF area of the insole is set to 14 mm, based on the benchmark. Moreover, an additional 18 mm is used as the reserved space for the following insole 3D modeling. Therefore, the height range of MF area can be determined, upper bound is 32 mm (14 mm + 18 mm), and lower bound is 18 mm (0 + 18 mm). As shown in the MF of Figure 3b, in order to make the surface of the area smoother, the height range of MF area is assigned different values at the similar interval (3/4 mm) according to measured pressure values. When the pressure value is higher, which indicates the abnormality of the characteristic point, then the height is assigned a lower value. Otherwise, the height is assigned a higher value. Figure 3 shows the process how pressure value is converted to height of each area.

The basic characteristics of the PPRI: (1) the fixed height was 18 mm (the thickness of the sole was 2 mm; (2) the insole was made for each subject in the same way; (3) the insole was made of Thermoplastic polyurethanes (TPU) 3D printing material, 30% filling degree, moderate hardness; (4) the surface of the insole was modeled by hundreds of characteristic points. 

### 2.3. Subjects and Methods

We used Gpower version 3.1.9.2 (University of Kiel, Kiel, Germany) to estimate a priori sample size, with a power of 80%, and an α level of 0.05. The expected effect size was calculated by the means (84.6, 103.5, 109.4) and similar standard deviation (19.5, 17.6, 21.5) of the pressure on the medial heel area under different insole conditions. The result revealed that the sample size of 10 subjects were sufficient for analysis. In order to ensure the degree of cooperation, we considered recruiting subjects who are younger than 30 years old and had no movement disorders except for flexible flatfeet (the orthopedic doctor would help us to judge). The recruitment target was mainly male, taking into account gender factors, some female subjects would also be recruited.

Finally, a total of 10 subjects with flexible flatfeet (8 males and 2 females) were recruited in this study to participate in the experiment. All subjects had no neurological diseases or any lower limb injuries that might affect natural gait behavior. Table 1 shows the demographic data of subjects. The AI was based on the static plantar pressure analysis of our plantar pressure test plate. The self-designed foot pressure plate can effectively confirm most sarapus in dynamic and static collection, the use of sensors has more advantages than traditional ink measurement methods to effectively evaluate ground reaction pressure, and has clinical application value [26]. All subjects signed a written informed consent before participating in the experiment. The gait tasks were performed indoors, on the same level.

**Figure 4:**
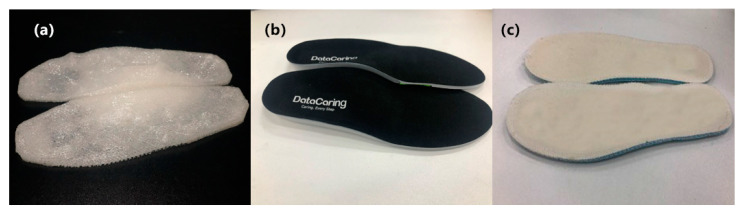
Comparison of different insoles (**a**) PPRI; (**b**) Orthotic insole; (**c**) Flat insole.

In order to control the variables, the shoes worn by the subjects in this experiment are all of the same brand style, and the specific size depended on the actual size of the subject. In this experiment, each subject performed three groups of experiments. The first group used ordinary flat insoles, the second group general orthotic insoles (Bio Orthotics, Bio-advanced, Langer, UK), and the third group their own PPRI. Wireless plantar pressure insoles were placed in the shoes for continuous monitoring of plantar pressure. Each experiment was carried out on the treadmill at low speed 0.8 m/s (2.88 km/h), normal speed 1.0 m/s (3.60 km/h), and fast 1.2 m/s (4.32 km/h). Before the start of each test, the subjects had 5 min to rest and 5 min to adapt the insole. All subjects walked in the normal heel-strike pattern. After the gait was stabilized, the continuous monitoring of plantar pressure data was started, and each test time lasted 1 min. We assumed that after the evaluation and comparison of the results, PPRI can improve the plantar pressure distribution of the sole and is suitable for adults with flexible flatfeet. Figure 4 shows the PPRI, the orthotic insole, and the flat insole.

### 2.4. Data Process

We used Xsensor FOOT and GAIT software (Xsensor, Calgary, AB, Canada) to analyze stance time, stride frequency, and peak pressure in each area of the sole and plantar contact area from the experimental gait data of trial. Stance time refers to the standing time of the gait cycle. Step frequency refers to the number of steps per minute in the analysis of gait experiment. In order to analyze the pressure changes in each area of the sole in more detail, we divided the M area pressure analysis into three parts and the H area pressure analysis into two parts. Therefore, we divided the foot into seven areas for data analysis: the toe (T), the first metatarsal (M1), the second to the third metatarsal (M2), the fourth to the fifth metatarsal (M3), midfoot (MF), medial heel (MH), and lateral heel (LH). We calculated the peak pressure of each area in the gait tasks. Considering the difference in the size of the subjects’ feet, we normalized the contact areas of T area, M area, MF area, and H area of each subject’s sole, and calculated the percentage of these four areas in the total contact area.

In addition, in order to reduce the degree of dispersion of the data, we averaged the data values of the left and right feet. The average value could be used to more intuitively notice the parameter changes in the experiment.

### 2.5. Statistical Analysis

We used Social Science Statistics Program 26.0 version (SPSS, Chicago, IL, USA) to analyze and process the experimental data. There were two sets of variables in the experimental data. Therefore, we used one-way analysis of variance (ANOVA) to compare gait parameters of three insoles worn by the subjects at each different gait speed. Levene’s test was used to test the homogeneity of the variances. A Kolmogorov–Smirnov test was used to evaluate the normality of the data, a Wilcoxon test was used when the data were not normally distributed. In the experimental analysis, null hypotheses of no difference were rejected if *p*-values were less than 0.05. Tukey was applied for post-testing data. Cohen’s f was used to measure the effect size (ES).

## 3. Results

### 3.1. Stance Time and Step Rate

Table 2 shows the comparison of stance time and step rate in different insoles at each gait speed, including the data of the subjects’ left and right feet and the average value of the feet, respectively.

In order to reduce the error caused by the difference between the left and right feet, we analyzed the average value. The main effect of the insole showed that the stance time of wearing PPRI insole was significantly lower than wearing the flat insole at fast gait speed (*p* = 0.008, ES = 0.53). Under other gait experimental conditions, there was no significant difference in stance time and step rate between insoles. In addition, as the gait speed grew, the stance time generally decreased and the gait frequency increased.

### 3.2. Peak Pressure

Table 3 shows the peak pressure comparison of wearing three experimental insoles at different gait speeds, including the data of the subjects’ left and right feet and the average value of the feet, respectively. 

**Figure 5:**
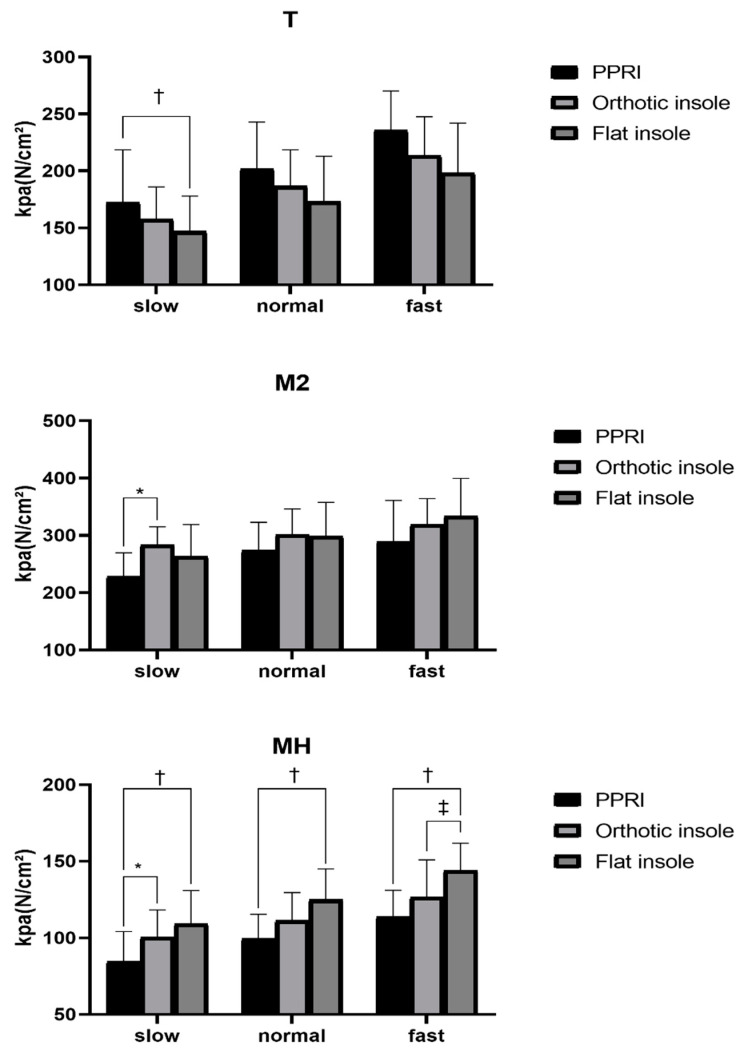
The peak pressure of the T, M2, and MH area. * significant difference found between the PPRI and orthotic insole. ^†^ significant difference found between the PPRI and flat insole. ^‡^ significant difference found between the orthotic insole and flat insole.

In order to reduce the error caused by the left and right feet, we analyzed the average peak pressure of each foot in each area. Figure 5 showed the peak pressure of the T, M2, and MH area, where reflected some significances. It is obvious that with the increase in gait speed, the pressure in each area of the sole shows an upward trend except the midfoot area.

In the T area, the peak pressure of wearing the PPRI was significantly greater than wearing the flat insole (*p* = 0.033, ES = 0.50) at slow gait speed. However, the pressure difference between wearing orthopedic insoles and wearing PPRI and flat insoles were not significant.

In the M2 area, the peak pressure in the PPRI insole was significantly lower than in the orthotic insole at slow gait speed (*p* = 0.009, ES = 0.64).

In the MH area, the peak pressure of the PPRI was significantly lower than the flat insole at slow (*p* = 0.009, ES = 0.66), normal (*p* = 0.004, ES = 0.70) and fast (*p* = 0.002, ES = 0.79) gait speeds, and was significantly lower than that of orthotic insoles at slow gait speed (*p* = 0.040, ES = 0.66). In addition, the peak pressure of orthotic insoles was significantly lower than that of flat insole during fast (*p* = 0.015, ES = 0.79) gait speed.

In the LH area, the peak pressure of wearing the PPRI was significantly lower than that of the flat insole at normal gait speed (*p* = 0.013, ES = 1.09). The peak pressure of wearing the orthotic insole was significantly lower than wearing the flat insole at slow (*p* = 0.004, ES = 0.81), normal (*p* < 0.001, ES = 1.09), fast gait speeds (*p* = 0.033, ES = 0.57).

In the multiple comparisons of the M1, M3, and MF areas of three insoles, there was no significant difference found between the insoles at each gait speed.

### 3.3. Contact Area

In this study, we divided the plantar contact area into four parts: toe area (T), metatarsal area (M), midfoot area (MF), and hindfoot area (H). Table 4 shows the comparison the percentage of each area when wearing different insole at three gait speeds. 

The results of multiple comparisons in the T area of three insoles were similar to the M1, M3, and MF areas.

In the M area, the contact area of the PPRI was lower than the flat insole at slow gait speed (*p* = 0.003, ES = 0.93) and normal gait speed (*p* = 0.026, ES = 0.49).

In the MF area, the contact area of the PPRI (*p* = 0.018, ES = 1.08) and the orthotic insole (*p* = 0.001, ES = 1.08) was higher than the flat insole at slow gait speed.

In the H area, the contact area of the PPRI was higher than the flat insole at slow (*p* = 0.001, ES = 1.31), normal (*p* < 0.001, ES = 1.54), and fast gait speeds (*p* = 0.001, ES = 1.14). the contact area of the orthotic insole was higher than the flat insole at slow (*p* < 0.001, ES = 1.31), normal (*p* < 0.001, ES = 1.54), and fast gait speed (*p* = 0.004, ES = 1.14).

## 4. Discussion

The present study showed that, compared to the flat insole, the stance time with the PPRI was reduced when level walking at slow gait speed (0.8 m/s). Huang et al. pointed in the research of prefabricated insoles that the shortening of stance time within the range of gait balance shows benefits for gait efficiency [17], which was consistent with the aforementioned results of this study. However, with the increase in gait speed, compared with the other two insoles, the stance time when wearing PPRI did not decrease and remained basically the same. The reduction in the stance time indicates that the gait efficiency of every patient was improved when wearing the PPRI compared to the flat insole, and orthotic insole performs mediocrely. 

In the walking process of the experiment, all subjects used the heel-strike pattern. This is a walking mode in which the pressure center of the sole is gradually transferred from the hind foot part to the forefoot part through the midfoot force, and the forefoot is responsible for the propulsion [27]. Compared with wearing flat sole insoles, the peak pressure of T area of subjects wearing PPRI increased significantly. The toe of the forefoot plays an important role in the propulsion of the gait, the increase in peak pressure of the T area helped to push the foot forward.

As shown in the M2 part, in Figure 5, at the second to third metatarsal, the unloading ability of wearing the PPRI was better than the orthotic insole and flat insole, the peak pressure was reduced with all three kinds of gait speed, especially at the slow gait speed. What we can confirm was that wearing PPRI can significantly reduce the pressure of the second to third metatarsals, and the plantar pressure impacted in the forefoot area is most likely to redistribute from the main M2 area to the first metatarsal area and the rest of the toe area, which was very beneficial to improve the movement stability of individuals with flexible flatfeet [21]. MH part in Figure 5 showed that PPRI had a better ability to unload pressure on the medial heel than the other two control groups at slow gait speed, while orthotic insoles only showed similar pressure unloading characteristics as PPRI in the fast gait task.

During the walking of adults, the heel area generally suffers frequent high impact pressure on the ground after the swing time of the gait cycle [28]. Previous studies had shown that the area with higher plantar pressure was located in the second and third metatarsal, as well as heel areas [21,29], the use of prefabricated insoles designed for adults with flatfeet helped to disperse the higher pressure and distribute the pressure on each area of the sole more evenly and reasonably [30,31].

The results showed that in all speed gait tasks, compared with wearing flat insoles, when the subjects walked with PPRI and orthotic insoles, the proportion of the force on their hindfoot and midfoot to that on the whole sole increased significantly. Previous studies pointed out that such changes had some potential positive effects, such as ensuring the comfort of the soles and effectively protecting the soft tissues of the roots from damage [32]. 

There was a meaningful change in the proportion of contact area that occurred in the metatarsal area, present study showed that when wearing PPRI, the percentage of the metatarsal area in contact area was lower than that of the flat insole at slow and normal gait speed. In the PPRI group, experimental insole improved the hindfoot response to the impact generated by contact with the ground, while ensuring the uniform and reasonable pressure distribution in the metatarsal area, it effectively reduced the pain and discomfort caused by high impact.

The current study also found that, with the increase in gait speed, the peak pressure of each area of the sole had a corresponding increase, except for the MF area. Changes in these areas were consistent with the results of previous study, the plantar pressure distribution of sarapus was affected by gait speed, and the analysis of plantar pressure was different under asynchronous speed [33]. However, during the walking process of the individual with flexible flatfoot in the experiment wearing different insoles, the peak pressure in the MF area was relatively stable at slow, normal, and fast gait speed. It is speculated that this phenomenon may be related to the loss of the arch of the flexible flatfeet during walking.

Discussion on the difference between the three experimental insoles. First of all, the flat insole was different from general orthotic insole and PPRI insole, it was similar to a flat surface, which hardly changed the shape of the subject’s sole. Secondly, the common point of orthotic insole and PPRI was that they both raised the arch, but orthotic insole would have a certain effect on the changes in the biomechanics of the soles, different subjects would have relatively large deviations, and PPRI made according to the subject’s sole pressure data could make the best possible use effect for every subject.

At present, our research aims to explore a design scheme of sole pressure redistribution insole that can be applied to the vast majority of patients with flexible flatfoot. However, due to the small number of experiments and the limited simulated scenes (only walking on flat ground), is the best design scheme determined under the current conditions the best design scheme among all patients with flexible flatfoot. We will add more subjects and walking tasks, including uphill or downhill, to further improve the design. We may monitor the energy consumption of subjects to evaluate the impact of different insoles on gait efficiency. 

## 5. Conclusions

Wearing individual customized insoles based on plantar pressure analysis is the most common method to help users change the abnormally distributed pressure on the pelma. However, previous studies were only divided into several plantar areas without detailed plantar characteristic analysis. This study designed the insoles PPRI that redistributes pressure based on the characteristic points of plantar pressure, and evaluated during walking of subjects while wearing the insoles. The test results show that the PPRI can improve the plantar pressure distribution and gait efficiency of adults with flexible flatfeet, and can be applied into clinical application.

## Figures and Tables

**Table 1:** Demographic data of subjects.

General Characteristics	Subjects (n = 10)
Number of subjects (Male/Female)	8/2
Height (cm)	167.9 ± 5.5
Body weight (kg)	67.4 ± 10.9
Foot length (cm)	25.4 ± 1.6
Ankle width (cm)	8.5 ± 0.6
Arch index (%)	31.7 ± 2.7

Values are expressed as mean ± SD.

**Table 2:** Comparison of stance time and step rate in different insoles at each gait speed.

	PPRI			Orthotic Insole			Flat Insole		
	Left	Right	Average	Left	Right	Average	Left	Right	Average
Stance time (ms)									
Slow ^†^	784.6 ± 64.8	793.9 ± 39.4	789.4 ± 51.0	803.9 ± 80.5	805.3 ± 75.4	804.6 ± 77.2	804.4 ± 74.6	819.5 ± 79.5	812.0 ± 76.3
normal	695.7 ± 45.9	699.2 ± 36.7	697.5 ± 38.5	696.6 ± 42.0	700.8 ± 33.9	698.7 ± 37.2	699.9 ± 50.4	707.5 ± 45.9	703.7 ± 47.4
fast	612.5 ± 43.5	622.4 ± s25.7	617.5 ± 32.9	638.0 ± 33.5	642.6 ± 28.7	639.8 ± 29.7	645.4 ± 33.8	668.2 ± 29.0	656.8 ± 29.7
Step rate (steps per minute)									
slow	94.7 ± 7.5	95.9 ± 5.2	95.3 ± 5.3	93.7 ± 6.5	94.7 ± 8.5	94.2 ± 7.1	94.0 ± 8.5	97.5 ± 11.3	95.8 ± 9.8
normal	104.6 ± 8.1	103.3 ± 6.8	104.0 ± 5.5	103.9 ± 5.4	105.2 ± 6.9	104.6 ± 5.5	104.6 ± 7.6	107.0 ± 12.7	105.8 ± 9.9
fast	115.4 ± 4.7	117.5 ± 7.5	116.5 ± 5.4	113.1 ± 4.9	114.3 ± 7.3	113.7 ± 5.5	111.7 ± 5.0	116.4 ± 8.3	114.1 ± 6.0

slow: 0.8 m/s, normal: 1.0 m/s, fast: 1.2 m/s. ^†^ significant difference found between the PPRI and flat insole.

**Table 3:** Comparison of peak pressure in different insoles at each gait speed.

	Ppri			Orthotic Insole			Flat Insole		
	Left	Right	Average	Left	Right	Average	Left	Right	Average
T (kpa)									
slow ^†^	179.7 ± 44.7	184.8 ± 73.3	182.2 ± 46.3	163.4 ± 41.8	152.1 ± 51.2	157.8 ± 28.2	141.3 ± 47.3	152.9 ± 66.7	147.0 ± 31.0
normal	203.1 ± 23.2	201.0 ± 72.5	202.0 ± 40.9	190.3 ± 40.4	183.2 ± 59.6	186.8 ± 31.8	164.6 ± 38.2	181.9 ± 74.7	173.3 ± 39.6
fast	233.6 ± 23.7	238.4 ± 75.8	236.0 ± 34.3	214.7 ± 33.2	213.2 ± 76.3	213.9 ± 33.8	188.9 ± 42.0	207.7 ± 84.3	198.3 ± 43.7
M1 (kpa)									
slow	158.8 ± 59.1	109.3 ± 31.0	134.0 ± 37.6	145.7 ± 37.3	103.0 ± 38.7	124.3 ± 25.3	146.7 ± 27.0	109.2 ± 31.9	128.0 ± 21.0
normal	174.5 ± 56.6	110.5 ± 33.4	142.5 ± 37.0	158.2 ± 36.7	111.1 ± 41.4	134.6 ± 29.1	153.2 ± 21.5	120.2 ± 35.0	136.7 ± 22.2
fast	196.8 ± 53.2	127.5 ± 30.7	162.2 ± 26.5	176.7 ± 25.8	126.8 ± 44.4	151.8 ± 25.3	165.8 ± 20.4	139.0 ± 41.6	152.4 ± 25.6
M2 (kpa)									
slow *	211.5 ± 42.7	246.1 ± 51.8	228.8 ± 40.9	285.6 ± 48.3	281.4 ± 57.6	283.5 ± 31.5	256.5 ± 53.4	271.2 ± 85.7	263.9 ± 55.1
normal	263.0 ± 47.5	284.1 ± 64.0	273.6 ± 49.1	305.3 ± 30.2	298.1 ± 88.0	301.7 ± 44.5	288.8 ± 40.2	307.6 ± 96.9	298.2 ± 59.5
fast	282.0 ± 60.5	290.8 ± 101.1	286.4 ± 71.5	310.3 ± 24.9	328.1 ± 82.3	319.2 ± 45.0	319.0 ± 39.7	348.3 ± 110.4	333.6 ± 65.8
M3 (kpa)									
slow	132.6 ± 36.3	140.7 ± 46.9	136.6 ± 38.2	153.9 ± 56.7	152.2 ± 42.5	153.1 ± 43.6	150.7 ± 51.2	141.0 ± 54.0	145.8 ± 51.0
normal	156.7 ± 48.8	156.4 ± 57.9	156.5 ± 48.5	172.9 ± 69.3	154.5 ± 55.6	163.7 ± 60.2	171.1 ± 69.6	154.7 ± 57.5	162.9 ± 62.5
fast	159.0 ± 50.8	153.5 ± 60.0	156.3 ± 52.5	173.9 ± 69.4	161.3 ± 66.1	167.6 ± 65.5	180.2 ± 77.3	156.1 ± 63.4	168.1 ± 69.1
MF (kpa)									
slow	80.9 ± 21.2	85.4 ± 11.7	83.1 ± 15.6	81.3 ± 13.6	87.3 ± 14.6	84.3 ± 12.9	80.2 ± 18.1	76.2 ± 14.9	78.2 ± 15.7
normal	75.0 ± 22.2	84.9 ± 10.9	79.9 ± 14.8	82.7 ± 13.7	84.4 ± 13.3	83.5 ± 12.5	84.0 ± 18.6	78.6 ± 19.0	81.3 ± 17.6
fast	71.7 ± 17.7	76.9 ± 17.9	74.3 ± 17.3	75.6 ± 11.7	82.2 ± 20.8	78.9 ± 14.2	85.7 ± 16.7	77.4 ± 17.1	81.6 ± 15.6
MH (kpa)									
slow *^,†^	92.6 ± 32.3	76.7 ± 11.3	84.6 ± 19.5	108.9 ± 28.8	98.2 ± 13.4	103.5 ± 17.6	110.4 ± 28.8	108.5 ± 15.0	109.4 ± 21.5
normal ^†^	106.7 ± 28.2	92.4 ± 12.2	99.6 ± 15.7	114.8 ± 23.1	108.0 ± 18.9	111.4 ± 18.1	126.0 ± 24.7	123.8 ± 16.0	124.9 ± 20.0
fast ^†,‡^	123.5 ± 28.1	103.9 ± 19.9	113.7 ± 17.3	128.4 ± 25.0	112.9 ± 30.1	120.6 ± 24.3	146.8 ± 24.6	141.2 ± 15.3	144.0 ± 17.8
LH (kpa)									
slow	116.8 ± 26.8	111.7 ± 15.5	114.3 ± 17.0	113.9 ± 28.6	93.7 ± 6.9	103.8 ± 15.8	130.0 ± 21.6	129.8 ± 12.3	129.9 ± 15.7
normal	125.4 ± 18.1	126.9 ± 23.6	126.2 ± 13.4	124.6 ± 23.8	105.4 ± 9.3	115.0 ± 13.9	145.1 ± 20.7	145.2 ± 13.3	145.1 ± 14.4
fast	144.4 ± 16.1	142.9 ± 32.1	143.7 ± 21.2	141.0 ± 28.4	116.4 ± 16.9	128.7 ± 19.2	166.6 ± 21.2	159.0 ± 22.0	162.8 ± 18.0

slow: 0.8 m/s, normal: 1.0 m/s, fast: 1.2 m/s. * significant difference found between the PPRI and orthotic insole. ^†^ significant difference found between the PPRI and flat insole. ^‡^ significant difference found between the orthotic insole and flat insole. T = toe, M1 = 1st metatarsal, M2 = 2–3rd metatarsal, M3 = 4–5th metatarsal, MF = midfoot, MH = medial foot, LH = lateral foot.

**Table 4:** Comparison of contact area.

	PPRI			Orthotic Insole			Flat Insole		
	Left	Right	Average	Left	Right	Average	Left	Right	Average
T (%)									
slow	9.3 ± 0.3	9.3 ± 0.7	9.3 ± 0.5	8.8 ± 0.4	8.7 ± 0.5	8.8 ± 0.5	9.4 ± 0.5	9.3 ± 0.7	9.4 ± 0.6
normal	9.3 ± 0.5	9.4 ± 0.5	9.4 ± 0.5	9.3 ± 0.5	8.9 ± 0.6	9.2 ± 0.6	9.4 ± 0.6	9.6 ± 0.7	9.5 ± 0.6
fast	9.3 ± 0.3	9.2 ± 0.4	9.3 ± 0.4	9.4 ± 0.6	9.3 ± 0.5	9.4 ± 0.5	9.5 ± 0.3	9.7 ± 0.5	9.6 ± 0.4
M (%)									
slow ^†^	41.4 ± 0.5	40.7 ± 0.6	41.0 ± 0.5	41.3 ± 0.4	41.6 ± 0.4	41.4 ± 0.4	41.8 ± 0.4	41.9 ± 0.6	41.8 ± 0.5
normal ^†^	41.6 ± 0.6	40.9 ± 0.4	41.2 ± 0.5	41.3 ± 0.3	41.9 ± 0.5	41.4 ± 0.4	41.9 ± 0.6	42.0 ± 0.6	41.9 ± 0.6
fast	41.6 ± 0.6	41.4 ± 0.3	41.5 ± 0.4	41.3 ± 0.5	41.5 ± 0.2	41.4 ± 0.3	41.9 ± 0.4	41.8 ± 0.6	41.8 ± 0.5
MF (%)									
slow ^†,‡^	27.6 ± 0.1	27.8 ± 0.2	27.7 ± 0.1	27.8 ± 0.3	27.9 ± 0.2	27.9 ± 0.2	27.3 ± 0,3	27.6 ± 0.2	27.4 ± 0.2
normal	27.5 ± 0.1	27.7 ± 0.1	27.6 ± 0.1	27.6 ± 0.2	27.6 ± 0.2	27.6 ± 0.2	27.4 ± 0.3	27.6 ± 0.1	27.5 ± 0.2
fast	27.4 ± 0.3	27.5 ± 0.2	27.5 ± 0.2	27.4 ± 0.1	27.5 ± 0.2	27.5 ± 0.1	27.3 ± 0.1	27.4 ± 0.2	27.3 ± 0.1
H (%)									
slow ^†,‡^	21.7 ± 0.3	22.1 ± 0.2	21.9 ± 0.2	22.0 ± 0.4	21.9 ± 0.3	22.0 ± 0.3	21.5 ± 0.3	21.1 ± 0.2	21.3 ± 0.2
normal ^†,‡^	21.6 ± 0.3	22.0 ± 0.1	21.8 ± 0.2	21.8 ± 0.3	21.8 ± 0.3	21.8 ± 0.3	21.4 ± 0.4	20.7 ± 0.3	21.1 ± 0.3
fast ^†,‡^	21.6 ± 0.1	21.8 ± 0.1	21.7 ± 0.1	21.8 ± 0.4	21.6 ± 0.2	21.7 ± 0.3	21.4 ± 0.3	21.1 ± 0.2	21.2 ± 0.3

^†^ significant difference found between the PPRI and flat insole. ^‡^ significant difference found between the orthotic insole and flat insole. T = toe, M = metatarsal, MF = midfoot, H = heel.

## Data Availability

The data presented in this study are available on request from the corresponding author. The data are not publicly available due to privacy.
